# Editors’ Note

**DOI:** 10.5195/ijt.2015.6188

**Published:** 2015-11-20

**Authors:** ELLEN R. COHN, JANA CASON

## Abstract

The Fall 2015 issue of the International Journal of Telerehabilitation (IJT) presents original articles in the areas of Research; Privacy and Security; and Technology Review. As of May 2015, the International Journal of Telerehabilitation (IJT) is live on PubMed Central: http://www.ncbi.nlm.nih.gov/pmc/journals/2411/. Articles from all past issues are indexed, as will be the current and future issues.

The Fall 2015 issue of the International Journal of Telerehabilitation (IJT) presents the original work of three distinguished and innovative interdisciplinary teams. Interdisciplinarity is also a hallmark of the IJT editorial staff, reviewers, and publishing team.

The first article is a product of engineers with expertise in computing science (LoPresti and Simpson) and a medical speech-language pathologist (Jinks) who practices in the area of assistive technology. These authors reported upon the degree to which consumers are satisfied with the provision of telerehabilitation services for augmentative and alternative communication or alternative computer accessibility.

A second article, by Proffitt, (occupational therapist) and Lange, (a physiotherapist with expertise in the use of interactive video games and virtual reality technologies) demonstrated the feasibility of employing a 6-week, game-based, in-home telerehabilitation exercise program using the Microsoft Kinect® for individuals with chronic stroke.

Finally, the third article co-authored by Watzlaf (health information management, with degrees in public health and epidemiology), DeAlmeida (health information systems, with a degree in cell and molecular biology), Zhou (with degrees in computer science and physics, and expertise in mathematical modeling on health related topics, information integration, and comparative genomics), and Hartman (a reference librarian with a degree in chemistry, who collaborates with faculty in the health sciences and serves as a liaison to a school of health and rehabilitation sciences) describes a protocol to conduct systematic reviews of research in telerehabilitation, with the aim that IJT readers can ultimately apply this protocol to identify best practices in telerehabilitation.

## PUBMED CENTRAL INDEXING

The International Journal of Telerehabilitation (IJT) is live on PubMed Central: http://www.ncbi.nlm.nih.gov/pmc/journals/2411/. Articles from all past issues are indexed, as will be the current and future issues.

## CALL FOR SUBMISSIONS

The next volume of the International Journal of Telerehabilitation will be published in 2016. We cordially invite your submissions by February 2, 2016. IJT accepts original research, case studies, viewpoints, technology reviews, book reviews, and country reports that detail the current status of telerehabilitation. Our peer reviewers constitute a multi-disciplinary group, and include researchers and clinicians from each of the major rehabilitation disciplines, rehabilitation engineers, health information managers, information technologists, and others. We welcome new peer-reviewers and invite guest editors with ideas for special, thematically focused issues. The IJT publication team is agile and can add additional issues as warranted to ensure currency. Please contact Editor Ellen Cohn, PhD (ecohn@pitt.edu) or Senior Associate Editor Jana Cason (jcason@spalding.edu) if you are interested in contributing to a future issue.

Respectfully,

Ellen R. Cohn, PhD, CCC-SLP

IJT Editor

Jana Cason, DHS, OTR/L, FAOTA

Issue Co-Editor and IJT Senior Associate Editor

